# Hypothalamus-Pituitary-Adrenal Dysfunction in Cholestatic Liver Disease

**DOI:** 10.3389/fendo.2018.00660

**Published:** 2018-11-12

**Authors:** Anca D. Petrescu, Jessica Kain, Victoria Liere, Trace Heavener, Sharon DeMorrow

**Affiliations:** ^1^Department of Medical Physiology, Texas A&M Health Science Center College of Medicine, Temple, TX, United States; ^2^Department of Internal Medicine, Texas A&M Health Science Center College of Medicine, Temple, TX, United States; ^3^Department of Research Services, Central Texas Veterans Health Care System, Temple, TX, United States

**Keywords:** glucocorticoids, corticotropin releasing hormone (CRH), adrenocorticotropic hormone (ACTH), cholestasis, bile acids, circadian rhythm

## Abstract

The Hypothalamic-Pituitary-Adrenal (HPA) axis has an important role in maintaining the physiological homeostasis in relation to external and internal stimuli. The HPA axis dysfunctions were extensively studied in neuroendocrine disorders such as depression and chronic fatigue syndrome but less so in hepatic cholestasis, cirrhosis or other liver diseases. The HPA axis controls many functions of the liver through neuroendocrine forward signaling pathways as well as negative feedback mechanisms, in health and disease. This review describes cell and molecular mechanisms of liver and HPA axis physiology and pathology. Evidence is presented from clinical and experimental model studies, demonstrating that dysfunctions of HPA axis are correlated with liver cholestatic disorders. The functional interactions of HPA axis with the liver and immune system in cases of bacterial and viral infections are also discussed. Proinflammatory cytokines stimulate glucocorticoid (GC) release by adrenals but they also inhibit bile acid (BA) efflux from liver. Chronic hepatic inflammation leads to cholestasis and impaired GC metabolism in the liver, so that HPA axis becomes depressed. Recently discovered interactions of GC with self-oscillating transcription factors that generate circadian rhythms of gene expression in brain and liver, in the context of GC replacement therapies, are also outlined.

## Introduction

**Figure 1:**
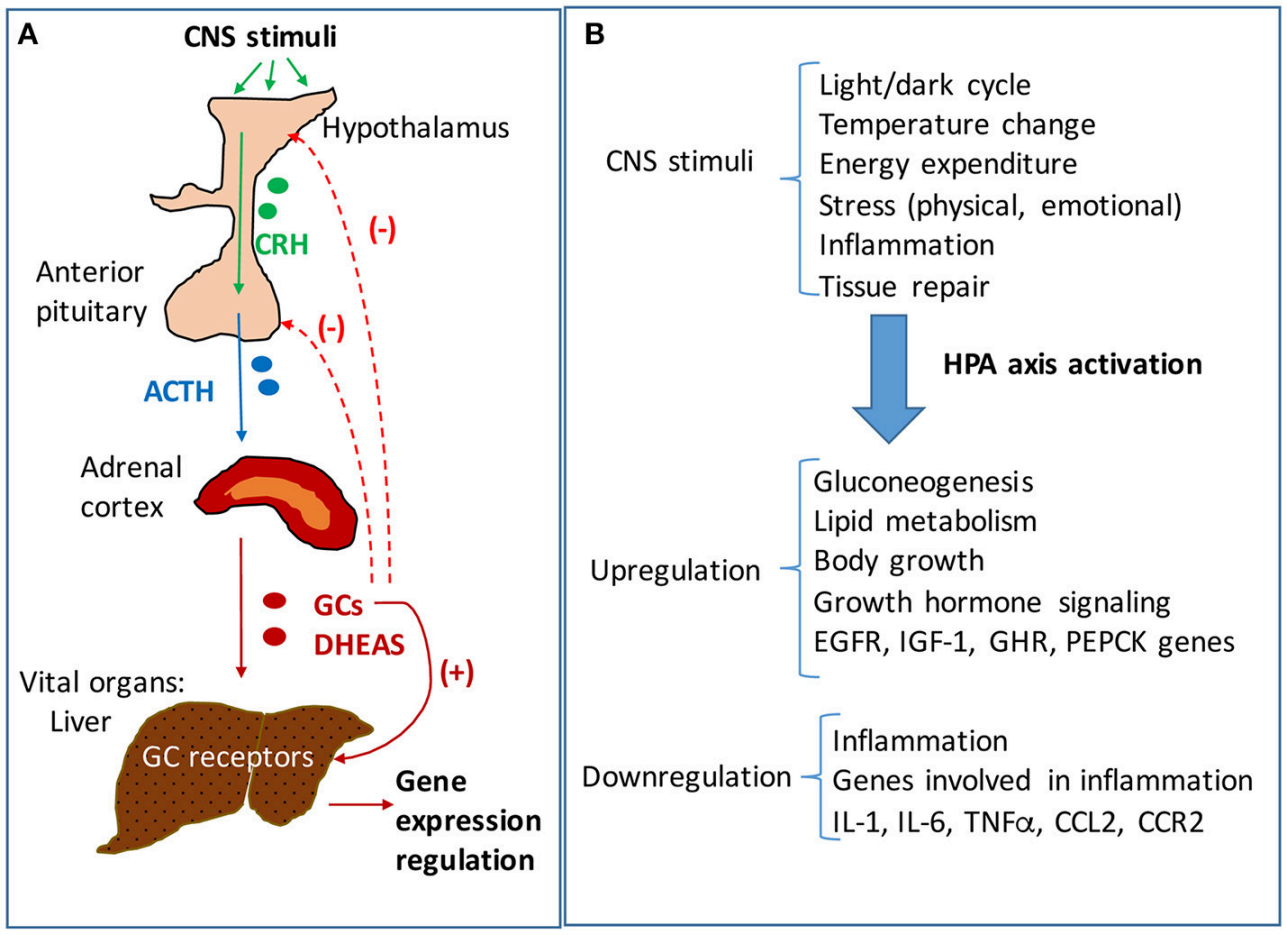
The hypothalamic-pituitary-adrenal (HPA) axis signaling and regulation of hepatic functions. **(A)** Neuroendocrine signaling pathway of HPA axis. Stimuli of central nervous system (CNS) result in activation of HPA axis signaling, starting with the hypothalamus' increased secretion of corticotropic releasing hormone (CRH). CRH activates the anterior pituitary gland to secrete adrenocorticotropic hormone (ACTH) which in turn stimulates the adrenal glands to produce glucocorticoids (GC) and androgens (DHEAS). The adrenal steroids reach vital organs such as the liver where they bind specific receptors with transcriptional functions. GC bind to glucocorticoid receptors (GR) which are highly expressed in liver, and modulate the expression of many genes involved in energy production and homeostasis, growth and inflammation. The liver is mainly regulated by HPA axis, but there are interactions of HPA axis with other hypothalamus-dependent signaling pathways. While GC stimulate downstream pathways, they also trigger a negative feedback loop, inhibiting CRH and ACTH production in hypothalamus and pituitary, respectively. **(B)** HPA axis regulation of liver functions in response to CNS stimuli. The liver controls the rate of energy fuel distribution to the body depending on HPA axis signaling. In periods of rest and relaxation the activity of HPA axis is low so that the liver stores glucose into glycogen and has a reduced rate of lipolysis directing the lipids for storage in the adipose. In contrast, in times of intense activities of the body when large amounts of energy are needed, the HPA axis is stimulated and results in signaling the liver to produce glucose and put it in blood circulation, to oxidize lipids and to enhance the rate of ATP production in the whole body.

Activation of hypothalamus-pituitary-adrenal (HPA) axis is an essential component of stress response driving the production of glucocorticoids (GC, cortisol in humans and corticosterone in rodents) necessary to mediate the adaptation to changes in environmental (e.g., temperature, toxic food, infections) and internal (inflammation, tissue damage) conditions. HPA axis is a complex neuroendocrine system that functions synergistically with the locus coeruleus/norepinephrine autonomic nervous system in response to stress and also in a circadian rhythm (1). Hypothalamic secretion of corticotropin releasing hormone (CRH) due to changes in physical, biochemical and/or physiological factors, causes activation of anterior pituitary gland which increases its release of adrenocorticotropic hormone (ACTH), which in turn stimulates adrenals to produce GC (Figure 1). Glucocorticoids are hormones that act not only in stressful situations but also continuously in circadian rhythms to regulate metabolic, cardiovascular and immunological homeostasis (1, 2). GC bind and activate specific nuclear receptors and upregulate the transcription of many genes involved in production of energy at a rate corresponding to stress conditions (3). In relation to HPA axis, GC function in a negative feedback loop inhibiting hypothalamic and pituitary functions and ensuring the suppression of their own activities at the end of the physiological crisis or of a circadian cycle (Figure 1).

The HPA axis dysfunctions were largely studied in relation to disorders such as depression, chronic fatigue syndrome (CFS) and fibromyalgia (FM), but less so in hepatic cholestasis, cirrhosis or other liver diseases (4). Because depression, CFS and FM often have the common symptoms of fatigue, asthenia, muscular weakness, which can get worse during stress, they have been correlated to an impaired stress response due to HPA axis dysfunction (5–8). Interestingly, clinical observations indicated that patients with cholestatic liver disease and primary biliary cirrhosis (PBC) have several associated systemic symptoms including fatigue and depression, with poorly understood etiology which may relate to dysfunctions of the HPA axis (9).

## Cell and molecular aspects of liver physiology

Liver is a vital organ with complex physiology and essential functions including: (i) regulation of glucose and lipid metabolism and homeostasis; (ii) bile acid (BA) synthesis, traffic to gallbladder, intestine, and reabsorption from the enterohepatic circulation into the liver; (iii) metabolic processing and excretion of xenobiotic toxicants and drugs; (iv) serum protein synthesis; (v) coagulation, fibrinolysis and platelet production.

An important physiologic role of the liver involves the synthesis of BA, formation, secretion and traffic of bile to the intestine, as well as reabsorption and recirculation of it throughout the enterohepatic circulation (10). Bile is a complex mixture of BA, phospholipids, cholesterol and aqueous components such as electrolytes (Na^+^, K^+^, HCO3^−^, ${\text{SO}}_{4}^{3-}$, ${\text{PO}}_{4}^{3-}$), bilirubin, amino acids, enzymes (alkaline and acid phosphatases), proteins and peptides (albumin, reduced and oxidized glutathione or GSH, GSSG) mixed in specific ratios (10, 11). The biliary tract is a major excretory route for toxic xenobiotic metabolites, and also for endogenous molecules such as cholesterol, bilirubin or BA which can become harmful when accumulated in excess in various tissues.

Hepatocytes, the parenchymal cells of the liver, and cholangiocytes, the epithelial cells lining the lumen of intrahepatic bile ducts, work together to produce and secrete bile. The BA biosynthetic pathways require 15 enzymes that modify the steroid ring, oxidize, cleave and conjugate the side chain of the molecule (12). Bile acids are synthetized in hepatocytes starting with oxidation of cholesterol by two different pathways. The classical or neutral one is initiated by microsomal cholesterol 7α-hydroxylase (CYP7A1) and produces both primary BA, i.e., cholic acid (CA) and chenodeoxycholic acid (CDCA). Alternatively, the acidic pathway starts with side chain hydroxylation by mitochondrial sterol-27 hydroxylase (CYP27A1) and produces only CDCA (12). The primary BA are then conjugated to glycine or taurine by BA-CoenzymeA: amino acid N-acetyl transferase (BAT) and their aqueous solubility is thus increased. Conjugated CA and CDCA are secreted into the bile ducts through membrane proteins named bile salt export pump (BSEP) (11, 12). Both hepatocytes and cholangiocytes face bile canaliculi through their apical membranes while connecting to each other through basolateral membranes (10). In the intestine, CA and CDCA are deconjugated, oxidized and dehydroxylated to form 7-deoxycholic and lithodeoxycholic acids by bacterial 7-α dehydroxylases (13). Most of intestinal BA are reabsorbed in the distal part of small intestine and transported back to the liver via enterohepatic circulation (14). Thus, BSEP transports BA from liver into the bile ducts, ASBT (apical sodium-dependent bile transporter or Slc10a2) and IBABP (ileal bile acid binding protein) facilitate the exit of BA from the intestine into the enterohepatic circulation, while NTCP (Na^+^-taurocholic acid co-transporting polypeptide or Slc10a1) mediates absorption of BA from blood circulation into the liver (15). Several genetic mutations of these proteins were identified in humans as being the cause of cholestatic diseases (Table 1).

**Table 1:** Genes associated with inborn hepatobiliary disorders and cholestasis.

**Gene**	**Function**	**Mutation-induced diseases**	**Serum and liver markers**	**References**
FXR/NR1H4	Senses BA and regulates transcription of genes with role in BA metabolism, transport, homeostasis	- Pregnancy induced cholestasis (PIC) - Idiopathic infantile cholestasis (IIC) - Progressive Familial Intrahepatic Cholestasis (PFIC)	Normal gGT High transaminases Ductular reaction	(16–18)
TGR5/GPBAR1	BA membrane receptor with role in biliary bicarbonate secretion; in intestine it induces FGF19 to reduce BA synthesis	- Primary sclerosing cholangitis (PSC)	Cholangiocyte proliferation	(19–22)
BSEP/ABCB11	BA efflux from liver into biliary tract	PFIC2 Benign recurrent intrahepatic cholestasis (BRIC2) DILI	Normal gGT High BA High liver transaminases Cholelithiasis due to poor BA sectretion into bile	(23, 24)
ASBT/SLC10A2	Bile absorption into small intestine	PFIC2 Primary BA malabsorption (PBAM)	Reduced plasma cholesterol	(25, 26)
NTCP/SLC10A1	BA uptake from enterohepatic circulation into the liver	PFIC1 ICP	High gGT in 30% of cases High BA High transaminases	(27–30)
TJP2	Structures tight junctions to form bile canaliculi	PFIC2 and 4	Normal gGT High BA Sclerosing cholangiatis	(27, 30)
ATP8B1	Phosphatidylserine translocase	PFIC1	Normal gGT High BA High transaminases	(31)
MDR3/ABCB4	Phosphatidylcholine flippase; role in forming micelles of BA with cholesterol and phospholipids in biliary canaliculi	PFIC1 PFIC3 Low PL-associated cholelithiasis (LPAC) ICP	High gGT in PFIC3 Normal BA High transaminases Ductular proliferation	(31–35)

Bile acid biosynthesis and enterohepatic circulation are regulated at several levels, mainly by transcriptional mechanisms (11, 36–38). Farnesoid-X receptor (FXR), a nuclear receptor activated by BA, regulates the transcription of genes involved in BA synthesis and transport mostly via small heterodimer partner (SHP). Thus, when activated by BA, FXR upregulates SHP expression. SHP is an atypical orphan nuclear receptor, does not have a DNA-binding domain and acts as a repressor by interacting with specific nuclear receptors such as HNF4α (hepatocyte nuclear factor 4-α), LXR (liver X receptor), FTF (fetoprotein transcription factor), to repress transcription of key genes with role in BA biosynthesis (e.g., cytochrome P proteins CYP7A1, CYP8B, CYP27A1), conjugation (VLACSR or very long chain acyl-CoA synthetase related gene; BAT or BA-CoA: amino acid N-acyltransferase) (39) and transport along the enterohepatic circulation (NTCP) (10, 12, 40). FXR was also found to upregulate genes that mediate bile efflux from the liver to biliary canaliculi, such as BSEP/ABCB11 and MDR3/ABCB4; FXR also stimulates the expression of OSTα and OSTβ (organic solute transporters) in basolateral membranes of hepatocytes and in ileal enterocytes, as well as intestinal expression of IBABP (intestinal bile acid-binding protein), mediating BA efflux from the liver or intestine to the portal vein (15). Thus, BA function as signaling molecules with specific affinity for FXR, regulating BA homeostasis. FXR was demonstrated to stimulate the expression of genes with role in liver protection against excessive concentrations of BA.

## Cholestasis etiology

Cholestasis is a hepatobiliary dysfunction characterized by reduced bile flow into the intestine, and accumulation of toxic bile components in the liver and outside the enterohepatic circulation, in various parts of the organism. The decreased bile secretion can be caused by a multitude of factors including: (i) impaired hepatocellular bile transport; (ii) bile duct pathology; (iii) bile flow obstructions due to gall stones or malignancies (41, 42). The metabolic consequences are usually categorized into systemic and intestinal effects. The systemic effects are caused by systemic accumulation of endogenous and exogenous compounds that normally would be excreted via the bile tract. The intestinal effects are a result of bile deficiency into the intestine, leading to malabsorption of lipid nutrients, steatorrhoea, malnutrition, impaired postprandial metabolism of chilomicrons, and also impaired intestinal BA-mediated signaling that can have systemic consequences (41).

Most forms of cholestasis are related to dysregulation of genes involved in bile secretion from liver into the biliary tract, or in bile transport from gallbladder to the intestine, or in the uptake of BA from enterohepatic circulation. Cholastasis and jaundice were found in both hereditary and acquired dysfunctions related to biliary production and circulation between liver and intestine. Hereditary hepatobiliary disorders characterized by cholestasis, include progressive familial intrahepatic cholestasis (PFIC) types 1–5, benign recurrent intrahepatic cholestasis (BRIC) 1–2, intrahepatic cholestasis of pregnancy (ICP), Low Phospholipid Associated Cholelithiasis or Gallbladder Disease 1. They are caused by defects in hepatobiliary transporters, structural proteins of canalicular ducts or in BA-sensing receptors such as FXR and TGR5 (13, 43). There are also differences and subgroups within these types of hereditary cholestatic diseases; for example, GGT enzyme or γ-glutamyl transferase is considered a cholestatic marker when increased in the serum, being associated with damage to apical membranes of bile ducts due to high BA concentrations in the bile. Higher than normal serum GGT was detected in PFIC3. However, there are forms of PFIC with cholestatic symptoms which do not present increased serum GGT, and where synthesis or BA secretion are virtually absent (13). Each type in each group of hereditary disorders is caused by loss of function mutations in specific genes encoding for: (i) membrane proteins with function in BA transport (see Table 1); (ii) proteins associated with bile canalicular duct structure; (iii) BA receptors and regulators of BA metabolism and enterohepatic circulation. Inheritance hypercolanemia such as PFIC2 and 4, as well as other forms of liver injuries including hepatocellular carcinoma (HCC) were found to be caused by mutations in tight junction protein 2 (TJP2) gene, also known as zona occludens-2 (27, 44, 45).

Interestingly, most of these diseases are treated successfully with corticosteroids together with statins, cholestyramine or ursodeoxycholic acid (UDCA), suggesting that stimulation of HPA axis can improve cholestatic conditions (13). Hereditary predisposition can also play a role in drug-induced intrahepatic cholestasis, such as hormonal contraceptive-induced cholestasis (13). FXR genetic polymorphism in humans is related to disease susceptibility and several clinical phenotypes including intrahepatic cholestasis of pregnancy (ICP) (16, 46).

The acquired cholestatic liver diseases include dysfunctions of hepatobiliary production and transport of the bile, caused by biliary duct obstruction due to gall stones or malignancies, or by medications and medical procedures such as liver transplant (13, 31).

## HPA axis regulates hepatic functions

**Figure 2:**
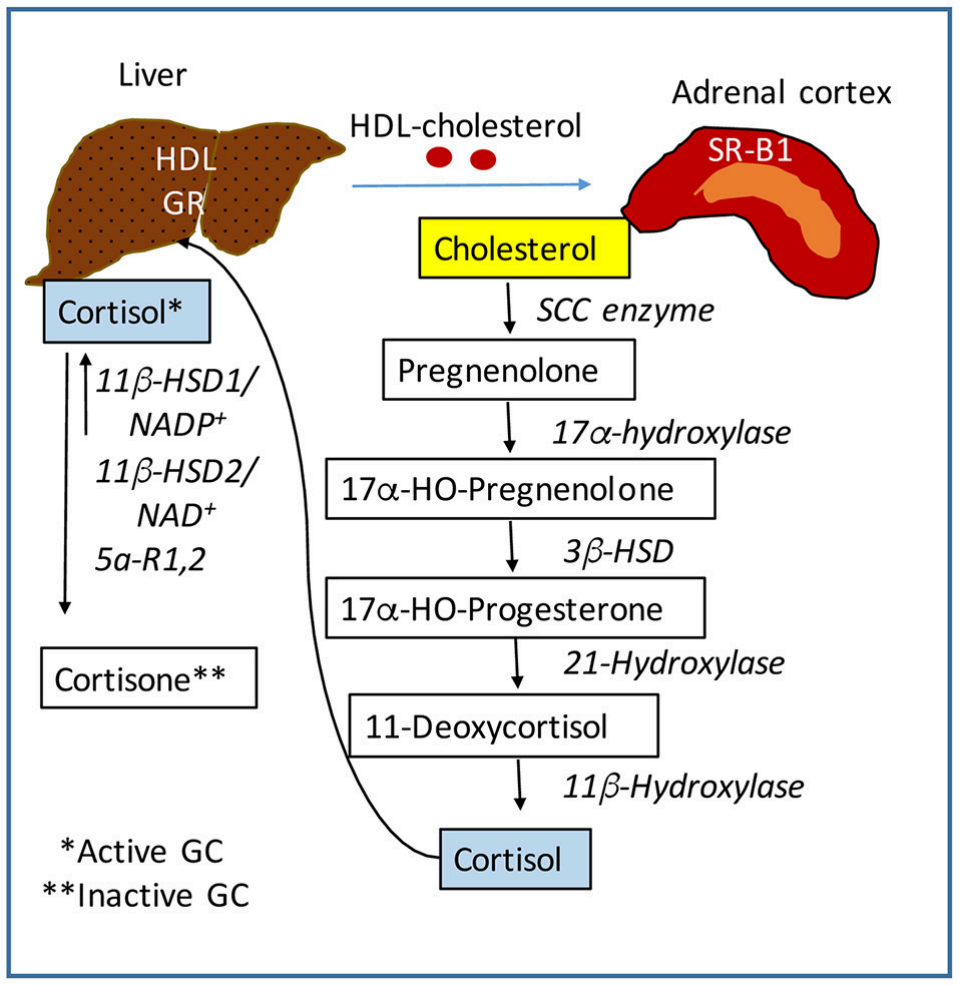
Regulation of cortisol activity. Cortisol is synthesized starting with cholesterol. The liver controls the level of lipids including cholesterol in the systemic circulation. The adrenal glands express scavenger receptor SR-B for high density lipoproteins (HDL) which mediate the uptake of cholesterol into the adrenal cortex. A series of steroidogenic enzymes in the inner mitochondrial membrane convert cholesterol into cortisol, the main GC with biological activity. Cortisol is released into the blood circulation and is taken up into the liver by GC receptors. Cortisol is converted into cortisone which is biologically inactive, by 11β-HSD (hydroxysteroid dehydrogenase) in the presence of NADP^+^. This form of 11β-HSD can also reduce cortisone to cortisol in the presence of NADPH. The NADP^+^ to NADPH ratio controls the activation of cortisol. SCC, side-chain cleavage enzyme. 3β-HSD, 11β-HSD, hydroxysteroid dehydrogenases.

The central nervous system (CNS) receives stimuli which are internal and external to the body, and in response sends signals to all organs to ensure metabolic and physiological homeostasis. The HPA axis controls many functions of the liver through neuroendocrine forward signaling pathways as well as negative feedback mechanisms as illustrated in Figure 1. The positive forward pathways start in the hypothalamus with secretion of CRH which stimulates the anterior pituitary gland to secrete ACTH which then stimulates the adrenals to release GC (Figure 1) (1, 3). Thus, according to the dark/light cycles, there is a diurnal cycle of cortisol release with a peak level in the morning and a gradual decrease during the day to the lowest level during night, in parallel with the energy necessities for high physical activity of the body during daytime and minimal activities during nighttime (1). Moreover, internal and external stressors such as illness, injuries and extreme physical conditions require additional effort of the body for survival, which involve even more stimulation of HPA axis. The regulation of hepatic functions by HPA axis has a component consisting of molecular mechanisms which inhibit HPA axis activity according to body's needs (47). Specifically, GC suppress HPA at hypothalamus and pituitary levels (Figure 1) (47, 48). The clearance of active forms of GC (cortisol, corticosterone) is critical because impaired GC clearing is associated with metabolic (glucose intolerance, hepatic steatosis) (49) and HPA suppression (2, 50). Figure 2 illustrates several steps in the metabolic processing of cortisol: (i) cholesterol which comes from liver via high density lipoprotein (HDL) particles is taken up by adrenal gland which expresses scavenger receptor type B1 (SR-B1) for HDL (51); (ii) cortisol is synthesized in the adrenal cortex starting with cholesterol as substrate for cholesterol side-chain cleavage enzyme (52); steroidogenic acute regulatory protein (StAR) is a mitochondrial protein with essential role in adrenal steroidogenesis (53); (iii) when released into the systemic circulation, cortisol reaches the liver, where it binds to GR (glucocorticoid receptors) and either activates or inhibits transcription of target genes; thus, cortisol stimulates gluconeogenesis (by increasing phosphoenolpyruvate carboxykinase or PEPCK gene expression), lipid metabolic pathways for increased growth and energy production and storage (insulin like growth factor 1 or IGF-1, epidermal growth receptor or EGFR, growth hormone receptor or GHR genes are transactivated by GC), while inhibiting genes involved in liver inflammation (such as interleukins IL-1, IL-6, C-C motif chemokine ligand 2 or CCL2, C-C chemokine receptor or CCR2) (54). Interestingly, in a gene profiling study on immune cells by microarray analysis, it was found that GC can have simultaneous inhibitory and stimulatory effects on inflammatory T helper subsets and apoptosis-related gene clusters, depending on the activation state of the cells (55). The concentration of cortisol is controlled at the local level in the liver by hydroxysteroid dehydrogenases (HSD) such as 11 β-HSD1 and 2 (53, 56). The 34 kD type 1 enzyme 11β-HSD1, catalyzes both the oxidation of cortisol with NADP^+^ as cofactor, and the reduction of cortisone using NADPH as cofactor and its activity direction is dictated by the NADP^+^/NADPH ratio. The 41 kD type 2 enzyme 11β-HSD2, has much higher affinity for cortisol than 11β-HSD1 and catalyzes only the oxidation of cortisol to cortisone using NAD^+^ cofactor. Loss of function mutations of 11β-HSD2 were correlated with hypercortisolism and congenital adrenal hyperplasia (57, 58) as well as metabolic syndrome (59). An alternative mechanism of cortisol regulation is via 5α-reductase (or 5α-R) an enzyme that catalyzes the A-ring reduction of pregnene steroids including GC (49). 5α-R1 and -R2 inactivate cortisol to 5α-dihydrocortisol, and their inhibition with dutasteride for example, resulted in insulin resistance and hepatic steatosis due to higher than normal rates of *de novo* lipogenesis (60). In an experimental study on the effect of 5α-R1 on the HPA axis activity, it was demonstrated that in 5α-R1 knockout mice, the hypothalamic hormones CRH and AVP (arginine vasopressin hormone) and the GC receptor Nr3c1 protein and mRNA were reduced as compared to control wild type mice (49). In conclusion, the levels of systemic cortisol and other biologically active GC are controlled by enzymes that degrade the active GC in vital tissues such as liver, kidney, brain.

## Clinical studies on HPA axis dysfunctions associated with hepatic cholestasis

Clinical reports indicate that cholestatic jaundice in infancy may be associated with hypoglycemia and GC deficiency due to various causes including dysfunctions of pituitary or adrenal glands. Isolated glucocorticoid deficiency (IGD) is a rare but potentially lethal hereditary disease caused by loss of function mutations of ACTH receptor gene (61). IGD-affected children have a deficient production of cortisol in the presence of markedly elevated ACTH levels and may also present with cholestatic symptoms such as jaundice, hyperpigmentation of the skin, hepatomegaly and increased levels of hepatic biomarkers in the serum (61, 62). Cholestasis secondary to panhypopituitarism in infants has also been reported (63–65). A retrospective case study of patients diagnosed with congenital hypopituitarism concluded that cholestatic jaundice was the major manifestation in 35% of patients in neonatal or infancy period (65). Infants with jaundice and cholestatic symptoms are usually screened for pituitary dysfunctions because growth hormone deficiency may affect bile duct formation (63). Since the congenital hypopituitarism is a relatively uncommon cause of neonatal cholestasis, little is known about the effect of anterior pituitary hormones on hepatic functions (66). In these cases the usual treatment is replacement of GC, growth hormone and thyroid hormones, which alleviate the cholestasis and hepatomegaly symptoms. Rare cases of pituitary stalk interruption syndrome (PSIS), characterized by several specific abnormalities of the brain detected by magnetic resonance imaging are associated with neonatal cholestasis (67). PSIS indicates a permanent deficiency of HPA axis. Cholestasis during the neonatal period is a frequent symptom of PSIS and it has been proposed to be used for early diagnosis of PSIS (67). A case study of an infant with congenital combined pituitary hormone deficiency (CCPHD) presenting cholestasis, focused on the histology of liver after hormone replacement therapy with growth hormone, thyroid hormone and hydrocortisone (68), and concluded that pituitary-mediated hormones, especially cortisol may be essential for the normal development of the bile ducts in neonates. Congenital isolated ACTH deficiency (IAD) is a disease characterized by low plasma ACTH and cortisol levels and preservation of all the other pituitary hormones (69). This disease is largely associated with mutations of TPIT, a T-box transcription factor with role in the differentiation of corticotroph lineage in humans and mice. A study on 91 IAD patients found prolonged neonatal jaundice in more than half due to cholestatic hepatitis. In most cases, the cholestasis was alleviated shortly with GC replacement (69). Even though numerous studies emphasized that neonate and infant liver cholestasis is often associated with hypocortisolism and is treated successfully by replacement therapy, the exact pathogenic mechanism of this type of cholestasis remains unclear.

Association of liver cholestasis and cirrhosis with dysfunctions of HPA axis was also reported in adult patients. Low levels of cortisol, indicating a dysfunction of the HPA axis, have been reported in patients with end-stage liver disease (70, 71). The diagnostic test for relative adrenal insufficiency in critically ill patients is having low serum cortisol concentrations even after ACTH administration. In a study on the adrenal function in patients with end-stage liver disease awaiting transplantation compared to healthy controls, the patients with liver disease had more than 60% reduction in plasma cortisol level in response to indirect adrenal stimulation via insulin-induced hypoglycemia, and a 39% decrease when ACTH was administered to induce cortisol production (72). Patients suffering with advanced liver diseases are usually treated with low doses of hydrocortisone to compensate for the adrenal deficiency (73, 74).

A cohort study of 340 patients with liver disease who were admitted to a liver transplant intensive care unit, was done in order to detect and define a possible hepatoadrenal syndrome as a common clinical condition (75). Thus, 72% of patients with various liver disorders met the criteria for adrenal insufficiency, with 92% of them being patients who underwent recent liver transplantation. An important conclusion was that lower than normal serum HDL levels were associated with adrenal insufficiency, suggesting that liver dysfunction can lead to impaired synthesis of cortisol in the adrenal cortex. Cholesterol is the main precursor of cortisol biosynthesis, and experimental studies proved that HDL is the preferred cholesterol source for the adrenal gland (51), since its receptor, SR-B1 in mouse and its human homolog CLA-1 were found to be highly expressed in the adrenals (76–78). Apolipoprotein A1 (ApoA-1), the major component of HDL was demonstrated to be decreased in patients with cirrhosis, and also after liver transplantation when methylprednisolone given intraoperatively may have contributed to repressing the HPA axis (75). A decrease in serum cholesterol has been observed in advanced liver diseases (79), and low HDL-cholesterol levels have predicted mortality in cirrhotic patients (79, 80). It has been demonstrated that proinflammatory cytokines which are elevated in hepatic cholestasis inhibit cortisol biosynthesis (81). Cytokines can also cause a rapid fall in plasma cholesterol *in vivo* (82), and were demonstrated to decrease apolipoprotein production by hepatocytes *in vitro* (83). More recent studies revealed that HDL is an important inhibitor of inflammatory responses and its dysfunction contributes to serious complications experienced by patients with acute and chronic liver disorders (80).

HPA axis and the liver are interdependent and function together to ensure the homeostasis of many metabolic pathways. Clinical conditions in which glucocorticoids are produced in excessive amounts, have also been related to the development of hepatic disorders. Cushing's syndrome (CS) is a rare condition in which the endogenous cortisol secretion is excessive and out of the HPA axis control due to abnormal secretion of ACTH from a pituitary tumor (84). A clinical study of 50 patients with active CS, found 20% to be diagnosed with hepatic steatosis based on radiological assessment using computed tomography and blood test for markers of CS and liver function (85). There are also reports of highly increased levels of cortisol and BA in liver and serum, associated with Cushing's syndrome and obesity, suggesting that patients with excessive GC are predisposed for higher than normal systemic BA levels (86).

Corticosteroids play an important role in the management of many inflammatory conditions but their systemic long-term use is often associated with serious side effects leading to osteoporosis, osteonecrosis, HPA axis suppression, weight gain and even alterations of brain-related functions (87–89). Corticosteroids are successfully used to treat hepatic conditions such as liver transplant, various forms of hepatitis and primary biliary cirrhosis (90, 91). However, among serious potential risks associated with systemic long-term corticosteroids are related to CNS functions, since about 20% of patients receiving high doses of corticosteroids develop psychiatric conditions such as depression and psychosis (92, 93).

## Association of HPA axis dysregulation and liver disease in experimental models

When clinical observations indicated that patients with liver diseases such as hepatic cholestasis in infants with hypocortisolism, or cholestatic biliary disease and cirrhosis in adults with severe symptoms of fatigue, were associated with suppressed HPA axis functions, research studies started to explore a possible link of hepatic dysfunction and HPA axis dysregulation by using experimental models. In the early 1990's, Swain and his group studied the HPA axis in a model of hepatic cholestasis in rats undergoing bile duct ligation (BDL) and demonstrated that there is an impaired activation of the HPA axis in cholestatic animals in response to various types of stress including chemical (9), physical (94) or immuno-induced stress (95). Thus, it was determined that cholestasis is associated with a significant suppression of HPA axis' ability to respond to stress, due to lower than normal levels of hypothalamic CRH and circulating ACTH in cholestatic animals. Interestingly, it was demonstrated that proinflammatory mediators such as cytokine IL-1, which are increased in blood circulation in hepatic cholestasis, suppressed ACTH release into the plasma of cholestatic rats and reduced the secretion of hypothalamic prostaglandin E2 as compared to sham-operated rats (95). More recent studies in our lab described new molecular mechanisms underlining the interplay between HPA axis and liver in hepatic cholestasis. The HPA axis function was assessed in various models of cholestasis including BDL, partial BDL and α-naphtylisothiocyanate (ANIT) intoxication of rats (96). The HPA activity reflected in the levels of tissue and circulating CRH, ACTH as well as corticosterone and cortisol indicated significant suppression of the HPA axis activities in all tested models. A novel finding of this study was that this suppression was associated with increased proliferation of cholangiocytes, the epithelial cells that line the bile ducts. Moreover, adrenalectomy or knockdown of hypothalamic CRH also induced cholangiocyte proliferation, while treatments with ACTH (systemically), CRH (centrally) or cortisol (systemically) after BDL-induced cholestasis, reduced the biliary mass as well as cholangiocyte proliferation (96). Assessment of the effect of low-dose corticosterone on chronic liver cholestasis in Mdr2^−/−^ mice (an alternative model of cholestatic disease) indicated that the increased intrahepatic bile duct mass, cholangiocyte proliferation and the overall liver fibrosis were significantly reduced by corticosterone treatment, with better results in males than in females (54).

A strong correlation of the liver disease-related fatigue with an impaired function of the HPA axis was also demonstrated by studies of locomotive behaviors of cholestatic rats (97, 98). Animals with BDL-induced cholestasis had reduced hypothalamic CRH levels and enhanced sensitivity to centrally infused CRH (97, 98). The locomotor activity was measured and used as an indicator of fatigue, showing that cholestatic rats had significantly lower basal locomotor activity compared to sham controls, and intracerebroventricular administration of CRH resulted in a large increase in the locomotor activity of BDL-rats (97). These results are in line with clinical studies indicating that PBC patients have an enhanced ACTH release after intravenous CRH infusion due to activation of the pituitary gland possibly explained by an upregulation of CRH type 1 receptor after CRH treatment (98).

Several studies on experimental animals showed evidence that glucocorticoids can also have detrimental effects on the liver functions. Thus, Lu et al. (86) demonstrated that administration of dexamethasone promoted cholestasis and increased BA in wild type mice but not in FXR-knockout mice. This study revealed that GC-activated GR repressed SHP expression by recruiting CtBP (C-terminal binding protein), a co-repressor that attenuates FXR transactivation of SHP gene. *In vitro* experiments proved that dexamethasone increased BA synthesis in cultured rat hepatocytes by induction of cholesterol 7-α hydroxylase (99).

## Crosstalk of HPA axis, liver and immune system

The physiological functions of peripheral organs are controlled by CNS through neuroendocrine signaling pathways, and the liver physiology is regulated by CNS mainly via the HPA axis. Moreover, in hepatic diseases, the communications of the injured liver with the brain can be affected by interfering mediators resulted from immune system activation and response to liver injuries. Studies on monocyte-derived or recombinant interleukin-1 (IL-1) for example, demonstrated that IL-1 was able to induce secretion of hormones within the HPA axis acting as immunoregulator with role in signaling between the immune and neuroendocrine systems (100). Numerous research reports emphasized that cytokines such as IL-1β, IL-6, IL-17, tumor necrosis factor-α (TNF-α), interferon-γ (IFN-γ), C-reactive protein (CRP) were increased in liver and serum of patients with cholestatic diseases (101, 102). Clinical research results were supported by data from experimental models which demonstrated elevated levels of the same proinflammatory cytokines in serum of animals with induced hepatic cholestasis (103–105). Studies in our lab demonstrated that IL-6, TNF-α and CCL2 (C chemokine ligand 2) were significantly elevated in the livers of Mdr2^−/−^ mice as compared to wild type controls (54). Interestingly, a sex disparity was observed in regard to the expression of these cytokines, some of them being more prevalent in females than in males. When the effect of corticosterone on the hepatic level of these cytokines was assessed, data showed that this adrenal GC was effective in reducing proinflammatory cytokine levels in males more than in females, suggesting that an additional gonadal mediator may be involved.

Cytokines have multiple functions including signaling the initiation of the immune response to liver injury, with progression to proinflammatory phase for injury containment, followed by tissue repair during an anti-inflammatory phase (106). In hepatic cholestasis and cirrhosis, liver inflammation is recognized as being essential for ailment progression, indicating an impaired immune function associated with these disorders. Possible deficiencies in signaling pathways between liver and HPA axis, or the immune and HPA axis are still to be investigated. It is known that hepatic injuries which lead to cholestasis, cause residential macrophages (Kupffer cells) to become active and produce proinflammatory cytokines such as IL-1β, TNFα, CCL2 which can attract and recruit monocytes from blood circulation into the liver (106). Activated Kupffer cells as well as recruited macrophages secrete more profibrotic and mitogenic cytokines such as TGFβ1 and PDGF which further activate hepatic stellate cells (HSC) which start transdifferentiating into myofibroblasts (106). The recruited monocytes and macrophages secrete also cytokines which signal T-cells to become activated (107). This inflammation of the liver results in elevated levels of cytokines in the systemic circulation, influencing other organs including those involved in the HPA axis (95, 101). Research on inflammatory mediators causing changes in CNS and inducing symptoms of fatigue, showed that intravenous administration of cytokines or endotoxin in rodents resulted in altered neurotransmitter levels including those involved in generating fatigue (108–110). Thus, low doses of IL-6 and TNF-α increased plasma levels of corticosterone in mice as much as did exposure to physical stress (restraint). However, macrophage depletion by treatment with clodronate liposomes attenuated the circulating corticosterone changes induced by these cytokines (110), suggesting that macrophages themselves had a critical role in activating HPA axis in response to increased cytokines. However, how liver diseases affect these signaling pathways initiated by cytokines to the brain and HPA axis are still poorly understood and warrant further investigations.

Inflammation-induced cholestasis can be caused by LPS (lipopolysaccharide) and endotoxin released by bacteria in various microbial infections (111). In response to LPS which is cleared by the liver, Kupffer cell-produced proinflammatory cytokines activate membrane receptors of hepatocytes and cholangiocytes, resulting in altered BA transporter expression and function (112). Studies in rodents demonstrated that LPS directly or via cytokines, reduced the expression of NTCP and BSEP BA transporters in the canalicular and basolateral membranes of hepatocytes, leading to decreased export of BA from these cells, and impairment in bile formation and circulation (113–115). Similar and even more complex immuno-hepatic signaling occurs in viral infections (116). In the early stages of the proinflammatory response, the cytokines released into systemic circulation, activate receptors in cells of HPA axis, resulting in production of adrenal GC to counteract the negative effects of the infection (116). However, in chronic inflammation, the liver condition worsens due to impairment in bile formation, and consequently, the HPA axis function is suppressed. The liver influences both GC biosynthesis in adrenals (by providing cholesterol) and GC's clearing from systemic circulation (51). When the liver is affected by cholestasis, its interaction with HPA axis becomes dysfunctional, and usually the hepatic cholestasis is accompanied by HPA axis depression (50). Thus, an organism can efficiently fight against infections as long as there is a balanced interaction of the immune system with HPA axis and the liver.

## Effects of cytokine therapies on HPA axis and liver functions

During 1980's several immunomodulating therapies such as IL-2 and INF-α were considered for standard medical treatments of disorders including cancer (e.g., renal cell cancer, leukemias, malignant melanomas), infectious diseases (e.g., HIV, HCV) and neural degeneration (multiple sclerosis, amyotrophic lateral sclerosis) (117). However, preclinical studies demonstrated that some cytokines used in these therapies, can have negative side effects on CNS, particularly on the HPA axis. It has been suggested that IL-2 which is a potent modulator of neural and neuroendocrine functions, can readily penetrate the BBB and even impair its function leading to capillary leakage (118). Thus, it was reported that IL-2 increases the release of CRH, AVP and somatostatin from the hypothalamus, as well as ACTH from pituitary, while inhibiting growth hormone releasing hormone and growth hormone from the pituitary (118). In cholinergic neurons, IL-2 stimulates nitric oxide (NO) production; when NO reaches CRH-producing cells, it stimulates CRH release. Dysregulation of IL-2 can contribute to functional and pathological alterations in the brain and in the immune system (118). Changes in HPA axis activity may result in cytokine-induced imbalance in functions of the brain and of all the other organs downstream of the HPA axis (119, 120). Interestingly, several hepatic abnormalities have been detected in the early clinical trials of IL-2 therapy (121). Thus, hepatic symptoms associated with cholestasis and hyperbilirubinemia, such as jaundice and hepatomegaly were reported to occur, with serum bilirubin levels returning to normal after IL-2 discontinuation. Many patients complained of flu-like symptoms with low energy, fatigue and malaise, suggesting possible suppressed HPA activity (121).

IFN-α has been shown to be effective in the treatment of various forms of cancer and infectious diseases. INF-α has numerous neuroendocrine effects within the HPA axis where it exhibits concentration-dependent biphasic activation and inhibition of the HPA axis (122) (123). IFN-α has antigenic similarities with ACTH, γ-endorphin and human leukocyte interferon, which suggests that all these proteins may derive from a common precursor (123). IFN-α binds to brain opiate receptors, and its CNS activity may be reversed by opioid antagonists. Dysfunctions of the HPA axis via increased CRH secretion, and of the hypothalamus-pituitary-thyroid (HPT) axis have been associated with the pathophysiology of some major forms of depression (124). Unlike IL-2, IFN-α stimulates HPA axis activity and may have positive effects on liver physiology. While no negative side effects were reported on IFN-α therapies of cancer, experimental data suggested that interferon signaling via toll-like receptor 7 prevented cholestasis and hepatotoxin-induced liver fibrosis in a rodent model (125). Case reports also showed successful results when using intravenous interferon to prevent recurrence of cholestatic hepatitis C virus, during anhepatic phase of liver transplantation (126). Various forms of INF-α are used in combination with antiviral medications for the treatment of hepatitis E. The first case of hepatitis E, caused by HEV (hepatitis E virus) was detected in 2008 (127). HEV was thought to be the most common of enterically transmitted acute hepatitis in the undeveloped countries, but recently an increasing number of HEV infection cases were reported in organ transplant recipients, or other immunosuppressed patients in developed countries (127). Patients suffering of chronic hepatitis E (CHE) present not only jaundice, abdominal pain but also fatigue and asthenia which may indicate a suppression of HPA axis in addition to hepatic injury symptoms. Many CHE cases have been successfully treated with IFN-α in combination with ribavirin (127). Liver transplant recipients seem more likely to develop chronic HEV after an acute infection, and there is accelerated progression to advanced fibrosis and cirrhosis (128). The first line of treatment is considered a decrease in immunosuppression, while pegylated interferon combined with ribavirin is used as a second line of treatment for liver transplant recipients (128). In summary, due to the diverse effects of cytokines on the CNS, neuroendocrine and immune system, the immunomodulating therapies have to be rigorously tested and optimized in regard with dosage and duration of treatments before being considered for standard medical procedures.

## Role of bile acids (BA) in the pathology of hepatoadrenal syndrome

Glucocorticoids via their receptor GR in the liver have a critical role in maintaining the systemic BA homeostasis during fasting-feeding cycle by controlling BA biosynthesis, recycling and enterohepatic circulation (129). Studies showed that HPA axis impairment in humans, and liver-specific knockout of GR in mice, disrupted the normal BA systemic and enterohepatic distribution of BA (129). In mice with hepatic GR deficiency, BA uptake/transport was impaired due to reduced expression of a major basolateral BA transporter (Na^+^-taurocholate transport protein, or Ntcp/Slc10a1) resulting in cholesterol-gallstone development and disruption of systemic BA circulation (129).

**Figure 3:**
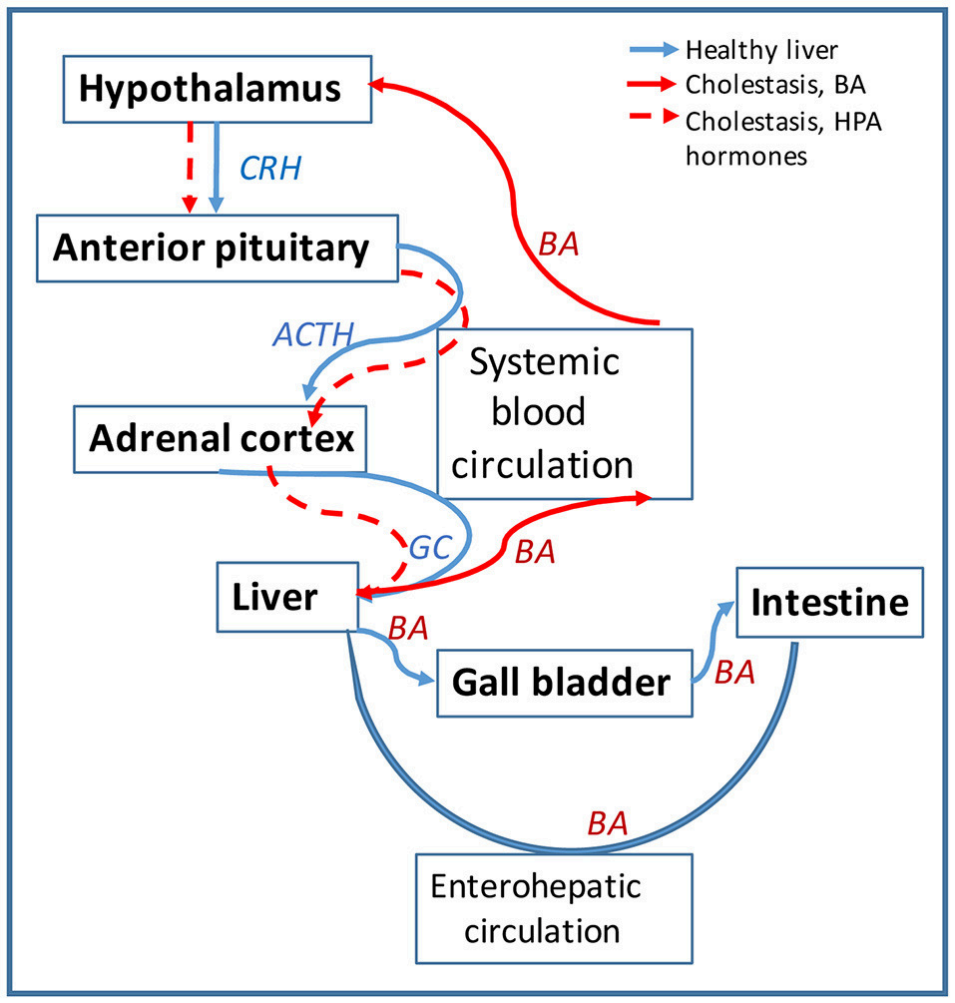
Bile acids (BA) affect the HPA axis function in hepatic cholestasis. Diagram showing the circulation of signaling hormones of HPA axis (CRH, ACTH and GC) and of BA produced in the liver is shown in blue arrows. Circulation of the same molecules is represented in red arrows for hepatic cholestasis. In healthy liver, BA are synthesized from cholesterol in hepatocytes and secreted via cholangiocytes into the gall bladder and then into intestine; most of BA is recycled from the intestine back into the liver via the enterohepatic circulation. In cholestatic liver, BA are not transported into the gall bladder but retained into the liver. BA escape into systemic circulation and cause leakage of blood brain barrier (BBB) getting entrance into the brain. In the hypothalamus, BA downregulate CHR and repress the HPA axis activity. The lack of GC in the liver alters BA homeostasis, causing BA to flood more into the systemic circulation.

BA are also signaling molecules that bind to specific nuclear receptors (NR) and modulate gene expression. The main BA-binding NR is FXR (farnesoid X receptor) which is activated by BA and downregulates genes involved in BA biosynthesis and transport (130, 131). BA can also activate signaling pathways via membrane G-protein coupled receptors such as TGR5 mediating cholangiocyte proliferation in hepatic cholestasis (132), or sphingosine-1-phosphate receptor 2 (S1PR2) inhibiting expression of enzymes of BA biosynthesis (133). During hepatic cholestasis, BA are accumulated in the liver and leaked into systemic circulation (50). BA and their receptors were detected in human and rodent brain (134, 135), but their function in CNS have only recently been investigated (135–138). Studies on BA as signaling molecules pointed to a direct involvement in the regulation of HPA axis. Thus, during cholestasis, serum BA levels are increased and are able to gain access to the brain via a leaky BBB (139) accumulating in hypothalamus (140). BA are transported into neurons via the apical sodium bile acid transporter (ASBT), and activate GR to suppress the hypothalamic expression of CRH and the entire HPA axis (140, 141). Furthermore, BA signaling has been shown to affect GC signaling directly in the liver during cholestasis, as illustrated in Figure 3. Specifically, BA inhibit the metabolism of cortisol in the liver by affecting key enzymes such as 5α-R1, 5α-R2, and 5β-reductase (2). *In vitro*, BA were potent competitive and transcriptional inhibitors of rat hepatic 5β-reductase (50). *In vivo*, it has been proved that increased systemic BA level induced by bile duct ligation, was associated with inhibition of 5β-reductase and down-regulation of HPA axis activity evidenced by reduced total daily production rates of GC (50). To confirm that alterations in corticosterone clearance can alter HPA activity, 5αR1 knockout (KO) mice were used (2). It was found that in the KO mice corticosterone clearance was defective, since its plasma levels were elevated in response to an acute bolus or a chronic infusion of corticosterone, as compared to controls (2). While there was no difference between deficient mice and controls in basal plasma levels of corticosterone, the 5αR1-KO mice exhibited relative adrenal insufficiency symptoms during stimulation by stress or ACTH (2). The corticosterone response was 26% lower after ACTH administration, 2.5 times lower after experiencing handling stress, and 43% lower after restraint stress in the KO mice when compared to the controls (2). Moreover, the KO mice had decreased mRNA levels of CRH, AVP and GR in the hypothalamus (2). These decreased levels confirmed that impaired peripheral clearance of GC caused HPA axis suppression in mice (2). Taken together, the current research shows that BA play a role in HPA activity modulation.

In regard with brain vs. serum concentrations of BA in hepatic cholestasis models, it was demonstrated that a very large (7–20-fold) increase of systemic BA concentration in rats with BDL-induced cholestasis, resulted in 25–30% increase in the brain BA content (139). In rats which were injected daily with BA until their serum BA concentration was 47.18 μM as compared to 22.58 μM in controls, BA caused significant BBB leakage by disrupting endothelial tight junctions via a Rac1-dependent mechanism (139). It was also demonstrated that BA accessed entry into neurons via ASBT and induced HPA-axis suppression by activating GR in neurons of hypothalamus of BDL-operated rats (140). Studies which aimed to do very accurate quantifications of specific BA in BDL-operated rats showed high correlations of brain and serum levels for CA, CDCA and DCA, and the highest brain-to-serum concentration ratios were found for the most hydrophobic BA (142). Older reports on the effect of BA on energy depleted BBB concluded that BA concentrations of 1.5 mM were actively opening the BBB (143). However, more recent work indicated that in cholestatic rats, increased serum BA resulted in BBB leakage (139, 140). Using LC/EIMS, Mano et al. detected protein-bound unconjugated BA in the brain of rats, the most abundant being CDCA in concentration of 1.6 nmol/g wet weight of brain tissue (134). In cholestasis, an additional factor besides the increased BA serum concentration, is the high level of proinflammatory cytokines in the systemic circulation. Similar inflammatory conditions such as experimental pancreatitis were shown to result in increased BBB permeability and were correlated with enhanced brain MCP-1 (monocyte chemoattracting protein-1) expression (144). Interestingly, several reports indicate that BA, especially DCA can be used as BBB permeability modifiers and mediate the uptake of very hydrophobic drugs such as gliclazide in animal models of diabetes (145).

## Circadian rhythms, HPA and liver diseases

Cortisol has a circadian rhythm with rising concentration in the early hours of the morning, peaking on waking and declining over the day with low concentrations in the evening and night (146). Once established after birth, the cortisol circadian rhythm is maintained throughout the childhood and adult life with only minimal alterations due to certain stressors such as jet lag, fatigue, etc. The HPA axis circadian rhythm is regulated by a central pacemaker in the suprachiasmatic nucleus in the hypothalamus (SCN), which requires a daily resetting via light/dark photoperiod to maintain a 24-h rhythm (146–148). It has been demonstrated GC pulses occur approximately every hour and a quarter following an additional ultradian rhythm of the HPA axis (149). In addition to this, it has been shown that several liver functions operate in circadian oscillations and that circadian clock proteins interact with proteins involved in liver function (150–153).

The circadian system consists of a central clock located in SCN, and peripheral clocks ubiquitously expressed in all tissues (1). The central clock receives light/dark signals from the eyes and sends communications in order to synchronize the activity of all tissues via their local clock systems (124). The main transcription factors with role in this process are CLOCK (a histone acetyltransferase) and its heterodimer BMAL1, which are self-oscillating transcription factors that generate circadian rhythms of gene expression in both CNS and peripheral tissues (154). CLOCK protein physically interacts with GRα and suppresses GR binding to GC-response elements on target genes, functioning as a reverse-phase negative regulator of GC-mediated pathways (154). Two circadian co-regulators, Cryptochromes 1 and 2 (CRY1, 2) are proteins that interact with GR in a ligand-dependent manner and repress GC-mediated gene transcription (155). GC are thus able to temporarily uncouple their target genes from the rhythm dictated by the central clock in CNS, and it is believed that this may be a protective effect of GC in stress response (1).

Since there are obvious links of HPA axis activity and circadian rhythm regulators with liver functions, the pathology in one system may lead to disruption in another. Many forms of hepatic cholestasis are treated with GC replacement therapy which has beneficial but also negative side effects in long term treatments. Future studies on the crosstalk of GR with clock proteins in the regulation of GC-controlled genes will improve GC-based therapies.

## Conclusions and future perspectives

In summary, clinical reports and experimental model studies demonstrate a strong association of cholestatic liver disorders with dysfunctions of the HPA axis. Several physiological functions connect the liver with the HPA axis: (i) a neuroendocrine signaling pathway along the HPA axis regulates the hepatic functions according to environmental and stress conditions; (ii) metabolic pathways of cholesterol synthesis in the liver, and cholesterol transport from liver to the adrenal glands for production of GC and other adrenal hormones, affect the HPA axis functions; (iii) the interaction of both liver and HPA axis with the immune system is critical for constant surveillance against injury factors, as well as initiation and progression of tissue repair; (iv) increase of bile acids in the systemic circulation due to liver cholestasis has a major impact on the brain and especially the HPA axis function. Any dysfunction of the liver, HPA or immune system can result in impaired function of the other connected systems. Due to this close interconnection, therapies of cholestatic liver diseases need to address and correct the HPA axis dysregulation in addition to solving the hepatic problems.

The interactions between HPA axis and the liver are very intricate and still not completely understood. Thus, aspects of liver dysfunction having effects on HPA axis and how therapies directed to the liver could help alleviate disorders related to HPA axis dysregulation, are still to be investigated (89). Also, more investigations on the cellular and molecular mechanisms triggered by cytokines in liver and HPA components, are needed for the development of better immunotherapies with large spectrum of activities (125, 126).

It was reported that despite elevated cortisol levels during critical illness, there is a tissue resistance to GC which is believed to occur due to insufficient GC-mediated anti-inflammatory response (156). This type of questions could be possibly answered by research on oscillatory activities in general that characterize the HPA axis and its signaling hormones, including GCs. New data demonstrate that various tissues including the brain, adrenal cortex and liver, are insensitive to continuous hormone stimulation, but very responsive to oscillating signals (157). More integration of the information coming from different areas of research on the HPA axis and the liver is needed for understanding the regulation of GC biosynthesis, plasma GC clearance and GC-signaling in acute stress and trauma.

## Author contributions

All authors listed have made a substantial, direct and intellectual contribution to the work, and approved it for publication.

### Conflict of interest statement

The authors declare that the research was conducted in the absence of any commercial or financial relationships that could be construed as a potential conflict of interest.
